# Unlocking ocular biomarkers for early detection of Alzheimer's disease

**DOI:** 10.1002/alz.14567

**Published:** 2025-02-19

**Authors:** Nikhil Dave, Melissa Lee, Hania Pavlou, Owen Im, Kim Goh, Sam Ulin, Kristina Malzbender, Eli Shobin, Aishwarya Sukumar

**Affiliations:** ^1^ Gates Ventures Kirkland Washington USA; ^2^ Alzheimer's Drug Discovery Foundation New York New York USA; ^3^ ClearView Healthcare Partners Newton Massachusetts USA

**Keywords:** Alzheimer's disease, hyperspectral imaging, non‐invasive screening, ocular biomarkers, ocular screening, optical coherence tomography

## Abstract

Recent evidence suggests that ocular testing holds promise as a non‐invasive and cost‐effective method for the early detection of Alzheimer's disease (AD). After interviews with neurologists, optometrists, primary care physicians, and ophthalmologists, the potential for ocular biomarker testing to become a standard clinical practice in the future was assessed. Ocular tests offer a non‐invasive alternative to blood‐based testing, capturing a substantial niche of ≈ 4 to 8 million individuals in the United States during routine eye exams. Technical requirements for broad adoption include high accuracy comparable to blood‐based tests and 510(k) clearance. Ocular biomarker technology must meet the practical requirements of optometrists and ophthalmologists, including ease of implementation, automation, and a clear path to profitability. A sufficient body of evidence to support guideline inclusion, reimbursement, and clinical actionability will facilitate the adoption. As the field evolves, advances such as earlier detection of preclinical AD may further expand the role of ocular testing.

**Highlights:**

- Ocular biomarkers offer another non‐invasive alternative to blood‐based Alzheimer's disease testing.
- Wide adoption will require accuracy akin to blood‐based tests and 510(k) clearance.
- Ocular screening could benefit ≈ 4 to 8 million US individuals conducting routine eye exams.
- Current ocular offerings remain nascent, and advances could expand this reach.
- New technology must show ease of implementation, automation, and a path to profit.

## 1 INTRODUCTION

Alzheimer's disease (AD) is the most prevalent form of dementia, currently affecting ≈ 7 million Americans ages ≥ 65.^1^, ^2^ This progressive neurodegenerative disorder is marked by cognitive decline and memory loss, with amyloid plaques and tau tangles as its primary hallmarks.^2^ Beyond the patients themselves, AD also imposes significant emotional, physical, and financial burdens on caregivers, families, and society at large.^2^ Early detection of AD can enable timely intervention with disease‐modifying treatments, improve patient outcomes, and help mitigate the broader societal impacts of the disease.^2^, ^3^, ^4^ Additionally, innovative diagnostic approaches are needed to identify AD in its preclinical stage, before symptoms appear.

Currently, a *post mortem* brain biopsy is the definitive method for confirming AD pathology. In clinical practice, amyloid positron emission tomography (PET) and cerebrospinal fluid (CSF) analysis are considered the gold standard for detecting amyloid pathology associated with AD. However, PET is costly and difficult to access, while CSF analysis is invasive and carries some risks (e.g., bleeding, infection).^5^, ^6^, ^7^ Consequently, primary care physicians (PCPs) and neurologists often rely on clinical evaluations, magnetic resonance imaging (MRI) or computed tomography (CT) scans, and cognitive assessments for differential diagnosis. A clinical diagnosis alone (i.e., clinical exam, cognitive testing, CT scan) that is conducted by PCPs has been shown to correctly support AD pathology in only ≈ 60% of cases.^8^ Similarly, with < 5% of AD cases being familial, genetic testing offers limited utility in identifying AD risk.^9^

Blood‐based testing for AD offers a cost‐effective, less invasive, and convenient alternative to current diagnostic methods. Promising blood‐based biomarkers (e.g., phosphorylated tau [‐tau]217) can detect amyloid beta (Aβ) and tau pathology before symptoms appear and may potentially supplant amyloid and/or tau PET scans in the future.^10^ Evidence for p‐tau217 is fast evolving: Quanterix's LucentAD pTau‐217 assay reports ≈ 89% sensitivity and ≈ 88% specificity^11^ and C_2_N Diagnostics’ PrecivityAD2, which combines both the Aβ42/40 ratio and p‐tau217/np‐tau217 ratio, reports ≈ 88% sensitivity and ≈ 89% specificity^12^ versus amyloid PET, which has traditionally shown ≈ 98% sensitivity and ≈ 80% specificity versus *post mortem* brain biopsy.^13^, ^14^, ^15^ While these developments highlight the potential of blood‐based biomarkers for streamlining AD diagnosis and reducing reliance on invasive tests, further studies are needed to confirm strong correlation with *post mortem* amyloid pathology. Additionally, blood‐based biomarkers are being used in clinical trials supporting the development of new treatments to identify preclinical AD patients (e.g., TRAILBLAZER‐ALZ 3, AHEAD 3‐45),^16^, ^17^ and will likely become standard for determining patient eligibility for novel preclinical therapies and evaluating treatment response.

Research into ocular biomarkers for AD diagnosis is ongoing, with early findings indicating potential for detecting AD biomarkers in the retina. Ocular biomarkers, such as retinal nerve fiber layer thickness, retinal ganglion cell loss, and retinal vascular changes, can identify AD before significant cognitive symptoms appear.^18^ Often regarded as the “window to the brain,” the retina is an extension of the nervous system with several direct connections to key brain regions. The retina also displays similarities to the brain's cellular composition, and several neurodegenerative disorders are known to have manifestations in the retina.^19^ Given that the retina is highly accessible via traditional ocular devices, it holds significant promise to be an AD biomarker.

Both blood‐based and ocular biomarkers could become transformative tools in the early detection of AD. However, considering the accessibility and performance of blood‐based tests, what will be the role of ocular biomarkers in the AD diagnostic paradigm? Here we present a perspective on the potential for ocular screening to become a standard clinical practice, including a review of the ocular technologies most likely to gain widespread use and the technical innovations and clinical evidence needed to support this approach.

## 2 METHODS

To inform this perspective on the potential of ocular screening for early detection of AD, in‐depth 60 minute interviews were conducted with a range of specialists. These included neurologists, ophthalmologists, and optometrists with expertise in AD diagnostics, a high volume of prescribing, or both. A total of 23 key opinion leaders (KOLs) were interviewed, comprising 5 expert neurologists, 5 general neurologists, 3 PCPs, 5 expert ophthalmologists, and 5 high‐volume optometrists/ophthalmologists.

All interviews were conducted virtually and in a double‐blind manner by ClearView Healthcare Partners. The key screening criteria to identify each type of interviewee were based on their expertise and are as follows:

- KOL neurologist: Must have ≥ 5 publications in the last 5 years related to AD diagnostics and must be a practicing physician with AD diagnostic clinical trial experience in the last 2 years.
- General neurologist: Must be a board‐certified neurologist with > 5 years of clinical experience and treat at least 100 AD patients per year.
- PCP: Must be a board‐certified PCP with > 5 years of clinical experience beyond training, who treats > 200 patients per month and has referred/diagnosed AD patients.
- Expert ophthalmologist: Must be a board‐certified ophthalmologist who has ≥ 5 peer‐reviewed publications in the last 5 years related to AD diagnostics and must be involved in purchasing decisions for diagnostic tools.
- High‐volume optometrist/ophthalmologist: Must be board certified, treat at least 50 patients per week, and be involved in purchasing decisions for diagnostic tools.

All interviewees were asked a series of questions within each of the following three categories: (1) the current AD testing landscape and its future evolution, (2) awareness and expectations of ocular testing, and (3) requirements necessary for adoption of ocular testing.

To assess interviewees’ perspectives on the present AD testing landscape, interviewees were asked to share their perception of current tools used for AD diagnosis, including blood‐based tests, across the following dimensions: performance, convenience, and cost/access. Additionally, participants were asked how they expect this landscape to evolve in the next 5 to 10 years, as well as what unmet needs they expect to persist.

To assess interviewees’ awareness of and expectations for ocular testing, participants were asked to share the value proposition they believe ocular technologies have in the screening and diagnosis of AD, as well as what they expect the role of ocular testing to be relative to blood‐based tests. Participants were then asked to describe segments of patients and segments of the optometrist and ophthalmologist markets that they believe would be more or less likely to adopt ocular tools.

To assess interviewees’ perspectives on the requirements for them to adopt ocular technologies in their own clinic, participants were asked to share their preferred imaging technologies and describe the required analytical performance (i.e., specificity, sensitivity), clinical evidence, and medical guidelines for them to adopt ocular technologies. Additionally, participants were asked to describe how the following operational factors might influence them to adopt ocular technologies in their clinical practice: upfront investment (i.e., cost, office space), required referral volume of relevant patients, and cost and reimbursement considerations.

Interview notes were collected by ClearView Healthcare Partners, and common themes among interviewees were extracted and synthesized. All descriptive statistics shared by participants were compiled and described as a range of values.

To further assess the potential of ocular screening for early detection of AD, the total addressable patient population was estimated across the following four use cases: (1) initial screening for preclinical AD in a general population, (2) use as a differential diagnostic tool for AD after a positive blood test in abnormal patient cases, (3) screening for preclinical AD in some segments of the general population referred for further workup, and (4) use in preventative treatment trials as a requirement after blood‐based diagnosis to confirm treatment eligibility. To estimate the total patient population in each use case, ClearView Healthcare Partners used a top‐down market sizing analysis, beginning with the total number of people in the United States between 55 to 85 years of age and then narrowing this population using a series of assumptions based either on publicly available data (e.g., a 2016 Harvard School of Public Health survey showed that 47% of Americans 65+ had interest in learning if they were likely to develop AD^20^) or data generated through KOL interviews (e.g., ≈ 40% of ophthalmologists and optometrists interviewed were receptive to screening for AD when there is no preclinical treatment available).

## 3 BIOMARKER TESTING IN THE FUTURE

### 3.1 Near term

Blood‐based tests are expected to become the standard modality for the detection of amyloid and/or tau pathology in the differential diagnosis of AD. Blood‐based tests currently have superior technical specifications (i.e., sensitivity and specificity) to ocular diagnostics. Additionally, simple blood draw is more commonly used in primary settings of care (i.e., where most patients first present) compared to ocular diagnostics. However, while specialized ocular equipment is less common in primary settings of care, automated screening systems have been implemented in this setting for some conditions (e.g., diabetic retinopathy screening systems). It is possible for PCPs and neurologists to similarly integrate the necessary equipment in their clinics if ocular technologies demonstrate acceptable sensitivity and specificity and are able to streamline clinical care.

In the near term, eye specialists may be reluctant to identify patients eligible for an AD ocular test based on cognitive symptoms, and testing of healthy individuals (i.e., in the absence of cognitive symptoms) is not expected to be recommended or widely adopted due to the limited clinical actionability or availability of preclinical treatments. Hence, while an out‐of‐pocket market driven by highly motivated individuals who regularly see an eye specialist and are interested in assessing their AD risk may exist, this market will be dependent on patient pull rather than physicians’ willingness to test, representing a total addressable population of 2.5 to 5 million adults in the United States.^21^, ^22^, ^23^

In practice, ophthalmologists and optometrists who have adopted AD biomarker testing are likely to offer the test to patients > 55 years of age during routine eye exams. Patients that elect to take the test are likely to be driven by their experience of AD in their family and/or their belief in lowering the risk of AD through lifestyle changes. If negative, patients may opt into AD ocular testing every 1 to 3 years, prompted by the eye specialist. If positive, ophthalmologists and optometrists will likely refer patients to their PCPs for continuous monitoring and guidance on lifestyle modifications. Future development of early AD symptoms will support further work‐up (e.g., blood test, cognitive tests, CT scan) and prompt referral to a neurologist.

### 3.2 Long term

**FIGURE 1:**
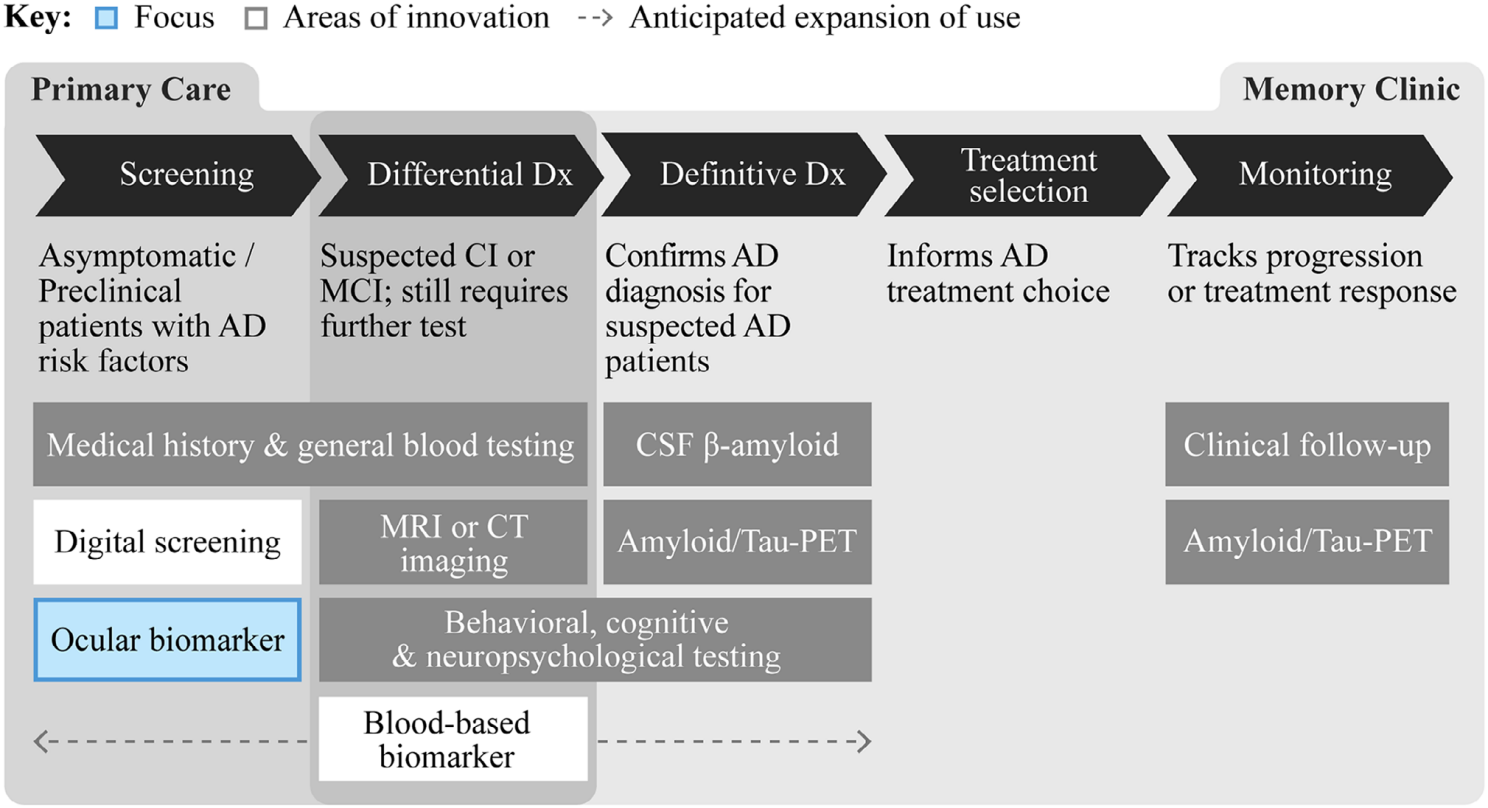
Potential future diagnostic paradigm for AD. In the current diagnostic paradigm for AD, digital tools are emerging for screening use cases, while blood‐based biomarkers are soon to become critical tools in differential diagnosis. Ocular technologies hold the most promise for the screening use case, to be used in tandem or potentially in place of digital screening tools. AD, Alzheimer's disease; CI, cognitive impairment; CSF, cerebrospinal fluid; CT, computed tomography; Dx, diagnosis; MCI, mild cognitive impairment; MRI, magnetic resonance tomography; PET, positron emission tomography.

The availability of preclinical AD treatments (i.e., AD prevention) in the long term may make screening the general population at risk of developing AD a standard practice. Ocular testing may find a niche in screening select patients during routine eye exams, while blood‐based tests become integrated into routine or annual physicals (Figure 1).

An estimated 4 to 8 million individuals, representing < 10% of the total addressable market for blood‐based testing, may be suitable for ocular AD screening.^21^, ^22^, ^23^ These patients might opt for ocular screening instead of, or in addition to, blood‐based tests under certain circumstances:

- Individuals who see an eye doctor more regularly than a PCP or have a stronger relationship with their eye doctor;
- Patients in underserved communities who visit community health clinics offering on‐site ocular screening;
- Needle‐averse individuals who decline blood tests and are referred to an optometrist or ophthalmologist by their PCP;^24^
- Those who, intentionally or unintentionally, undergo both screening methods.

## 4 PROMISING OCULAR TECHNOLOGIES

Various ocular technologies, including optical coherence tomography (OCT), hyperspectral imaging (HSI), and fluorescence imaging, have been explored for AD. Among these, OCT and HSI show the most promise based on current research, with each having unique advantages and limitations.^25^, ^26^, ^27^, ^28^ Other ocular measurement systems (e.g., eye tracking) were considered out of scope for the evaluation.

### 4.1 Optical coherence tomography

OCT is a widely used imaging technique that captures cross‐sectional images of the retina, allowing measurement of its layers.^28^ Most optometry and ophthalmology practices already use OCT, suggesting that a preclinical AD test via OCT could fit well into existing workflows.

For AD, OCT may be able to detect structural changes in the retina, choroid, and ganglion cell layers correlated with Aβ. For example, several studies have shown retinal nerve fiber layer thinning and thinning of the inner plexiform layer of the ganglion cell layer are associated with clinical mild cognitive impairment (MCI) and AD, as well as amyloid status.^29^, ^30^, ^31^, ^32^, ^33^, ^34^, ^35^, ^36^, ^37^, ^38^, ^39^, ^40^, ^41^, ^42^, ^43^, ^44^, ^45^, ^46^, ^47^, ^48^ Moreover, OCT's ability to measure neuroinflammation may offer a more specific AD biomarker and be more relevant upon validation of neuroinflammation‐targeting therapies.^18^, ^27^, ^28^ Current platforms have demonstrated 65% to 86% sensitivity and 55% to 75% specificity in patients with MCI, confirmed AD, and various other dementias.^18^, ^49^

While these studies are not definitive, they contribute to the large body of evidence suggesting that OCT examination of ocular structure changes holds significant potential in the diagnosis and screening of AD. However, it is important to emphasize that these changes can also indicate other neurodegenerative conditions like Huntington's disease and Parkinson's disease, as well as peripheral conditions including glaucoma, diabetes, and simply aging, raising concerns about specificity.^50^, ^51^ Further scientific research exploring the structural ocular changes specific to other neurodegenerative disease indications will be critical to improving specificity for OCT in AD.^25^, ^26^, ^28^

### 4.2 Hyperspectral imaging

HSI captures detailed spectral information from the retina, allowing for the identification of specific biomarkers like Aβ proteins. This technology uses multiple wavelengths of light to create a comprehensive image, or hypercube, that provides both spatial and spectral data.^25^, ^26^, ^52^

HSI's ability to detect retinal Aβ proteins offers a strong connection to AD pathology, making it a valuable diagnostic tool. However, HSI is still in its early stages, and most eye specialists are not very familiar with the technology, which presents greater barriers to integration into existing workflows. Current platforms have demonstrated ≈ 80% sensitivity and 60% to 85% specificity in patients with MCI or confirmed AD.^26^, ^53^

While OCT has clear workflow advantages and HSI has the potential to be more specific to AD, both technologies hold promise for early detection. Further validation through larger studies, the right patient population, and technological refinement are essential for widespread clinical adoption.

## 5 KEY REQUIREMENTS FOR OCULAR SCREENING IN AD

### 5.1 Market demand

For ocular screening to gain traction in the near term, there must be interest from adults aged 55+ in assessing their AD risk, despite the absence of preclinical treatments. This demographic likely includes health‐motivated individuals and those with a family history of AD. Current evidence on lifestyle modifications (e.g., diet, exercise) is significant but remains insufficient to prompt recommendations from key guidelines (e.g., American Academy of Neurology [AAN]). There are also substantial ethical concerns about creating worry in individuals who receive a positive result, particularly if they never develop AD and/or there is limited clinical actionability (i.e., due to lack of available, effective preclinical treatment). As such, the long‐term clinical utility of ocular screening hinges on the approval and reimbursement of a safe and effective preclinical AD therapy.

### 5.2 Workflow

Ocular technologies must be easy to implement; enable fast, non‐invasive, and repeatable imaging; and require minimal clinician or technician training (Table 1). OCT is advantageous for its fast imaging time (< 1 minute), non‐invasive nature, and widespread clinical use. While HSI can also be fast, HSI often necessitates eye dilation, lacks automation, and is not commonly used in clinics.

**TABLE 1:** Workflow, financial, and evidence requirements for ocular screening.

Workflow	
**Hardware**	Non‐invasive, non‐contact, strong repeatability and reliability, existing equipment, broader utility
**Software**	Minimal set‐up, seamless integration, results feed into EHR
**Process**	No manual calibration, no eye dilation, process < 5 minutes
**Training & support**	Minimal training required, easy‐to‐use by technicians, support services available

Financial	
**Cost**	Near term: $100–$200 Long term: Coverage by Medicare and major national plans
**Eye clinic profitability**	Break‐even in < 2 years

Evidence	
**Sensitivity**	90%–95%
**Specificity**	75%–85%
**Comparator**	Amyloid PET, tau PET, and/or prevailing blood‐based biomarkers (e.g., p‐tau‐217)
**Endorsement**	510(k) clearance, inclusion in AAN & AAO guidelines, supported by KOLs, neurology and ophthalmology communities

Abbreviations: AAO, American Academy of Ophthalmology; AAN, American Academy of Neurology; EHR, electronic health record; KOL, key opinion leader; PET, positron emission tomography; p‐tau, phosphorylated tau.

For AD risk analysis, OCT software can be integrated into existing systems or developed as standalone software, though integration may face challenges due to proprietary concerns. HSI can either use a separate hardware attachment with integrated software or a new standalone machine, although the latter may necessitate higher costs.

Beyond ease of implementation, ocular technologies will also need to establish connectivity to electronic health records for long‐term capture of results to support further work‐up, referrals, and ultimately improve patient care.

### 5.3 Financial

Ocular technologies will need to be profitable for ophthalmology and optometry clinics within 2 years of implementation. Limiting up‐front capital investment facilitates a faster break‐even point, favoring OCT technology. In the near term, elective AD screening will likely incur an out‐of‐pocket cost of $100to $200 (in line with current elective genetic tests), limiting the overall volume of tests conducted. However, insurance coverage would be expected upon the approval and reimbursement of preclinical AD therapies, supporting greater volume and overall profitability (Table 1).[Fig alz14567-fig-0002], [Fig alz14567-fig-0003]

### 5.4 Evidence

Ocular technologies used for screening must meet high sensitivity (90%–95%) and moderate specificity (75%–85%) standards. If a secondary screening tool is in place, such as blood‐based tests, physicians are likely to accept lower sensitivity (e.g., ≈ 85%) given secondary confirmation.

To drive adoption, ocular technologies must also demonstrate concordance with current approaches that detect AD pathology (e.g., amyloid PET, tau PET, or plasma p‐tau217), specifically in non‐symptomatic patients. US Food and Drug Administration 510(k) clearance and endorsement from both KOLs and neurology and ophthalmology communities will support inclusion in clinical guidelines (e.g., AAN and American Academy of Ophthalmology [AAO]) and widespread awareness and adoption of ocular biomarker testing (Table 1).

### 5.5 The road ahead

Today, the demand and performance of existing OCT and HSI technologies in the market fall short of the requirements for screening needed to compete with blood‐based testing. They currently focus on diagnostic applications rather than screening preclinical AD​. HSI particularly poses significant implementation and financial hurdles for adoption. The existence of preclinical AD therapy that drives physician demand for screening, together with significant technical improvement in screening for preclinical AD,​ can capture a small proportion (< 10%) of the blood‐based screening market.

## 6 WAYS TO UNLOCK FURTHER OPPORTUNITY FOR OCULAR BIOMARKERS

**FIGURE 2 alz14567-fig-0002:**
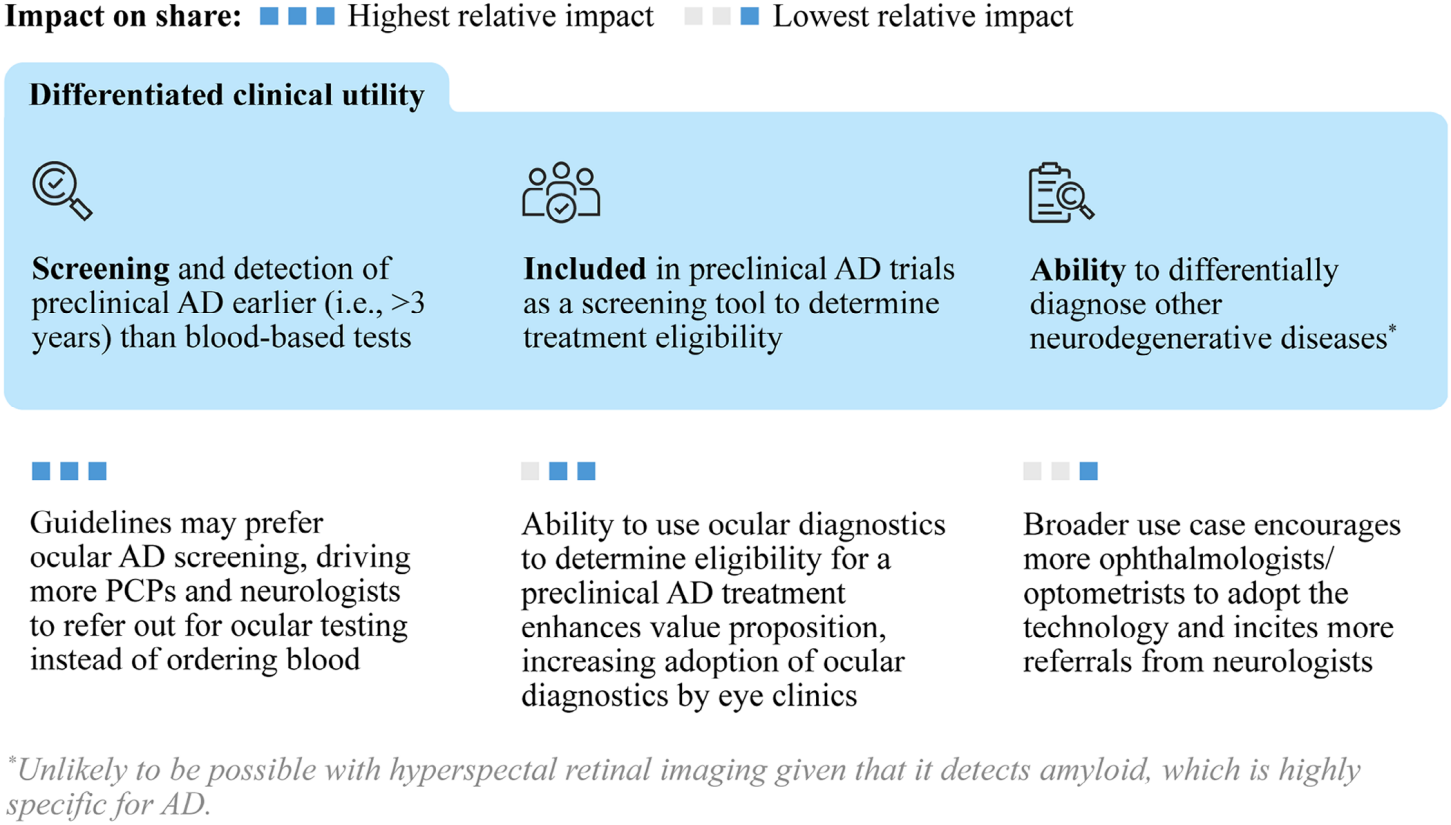
Ways to further unlock the opportunity for ocular biomarkers. If sufficient evidence demonstrates that ocular technologies can be used in early screening for preclinical AD, the opportunity for ocular biomarkers will increase significantly. Inclusion of ocular biomarkers as a screening tool for preclinical AD clinical trials will result in a modest increase in the ocular biomarker market, while evidence demonstrating ocular biomarkers as a tool for differential diagnosis of other neurodegenerative diseases will have a small but still relevant impact on the ocular biomarker market. AD, Alzheimer's disease; PCP, primary care provider.

Ocular technologies hold exciting potential to reach a larger market, beyond being an alternative screening method to blood for niche populations, through several key drivers of differentiation (Figure 2):

- Earlier detection of preclinical AD: If amyloid appears in detectable limits earlier in the retina, ocular biomarkers could potentially detect preclinical AD significantly earlier than blood tests. This would result in ocular screening being a preferred method for early detection, leading to more referrals from neurologists and PCPs.
- Preclinical treatment eligibility: Inclusion in preclinical AD trials to establish the clinical utility of ocular technologies in determining therapeutic eligibility would enhance the overall value proposition of ocular technology.
- Differential diagnosis of neurodegenerative diseases: Ocular technologies that have the potential to distinguish AD from other neurodegenerative diseases, such as Lewy body dementia or frontotemporal dementia, would increase the value proposition and utility of ocular diagnostics, and thus increase the willingness of eye clinics to adopt the technology.

**FIGURE 3 alz14567-fig-0003:**
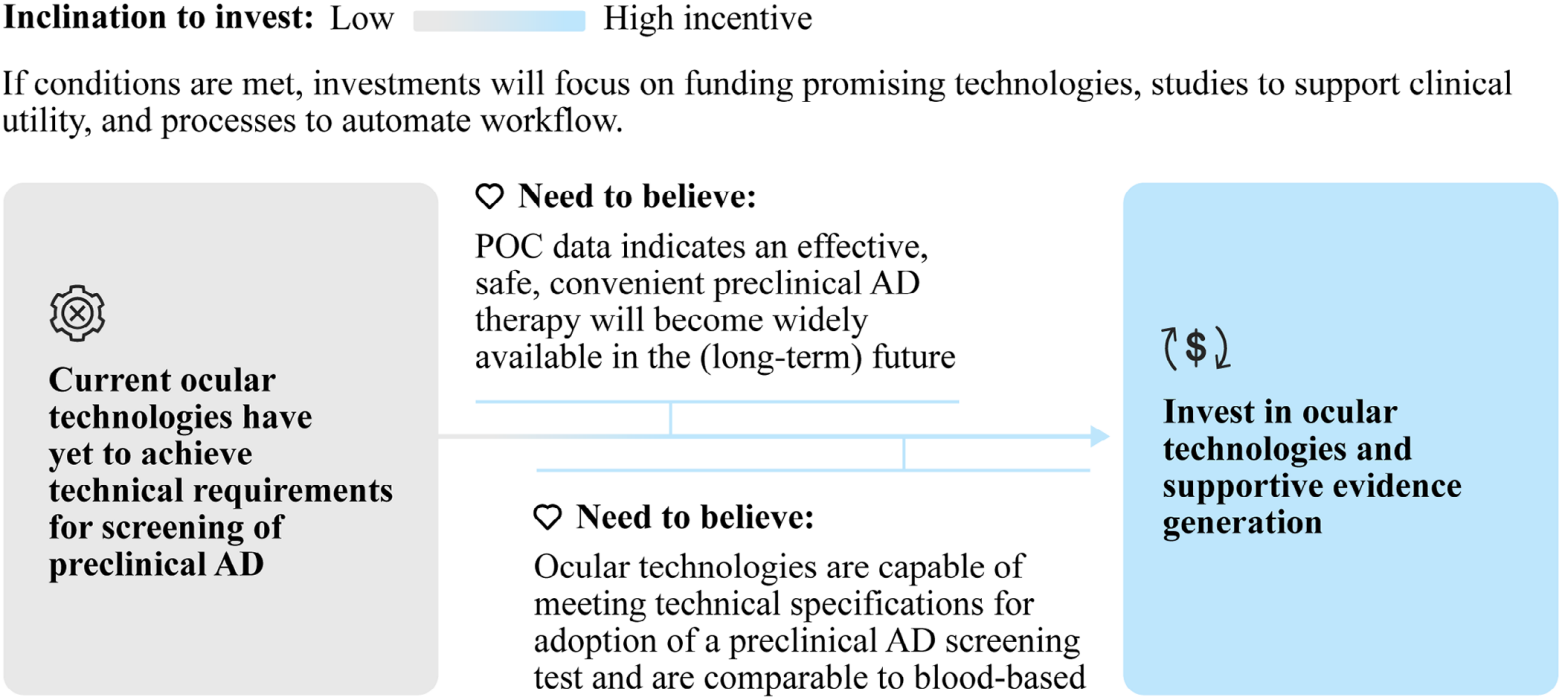
Illustrative path to investment for ocular technologies. Increased investment in ocular technologies will be driven by clinical trial evidence supporting preclinical AD therapies and evidence demonstrating that ocular technologies are capable of meeting technical specifications required for adoption. AD, Alzheimer's disease; POC, proof of concept.

Exploring these opportunities for differentiation may open a larger role for ocular biomarkers in advancing early detection and differential diagnosis of neurodegenerative diseases (Figure 3).

## 7 CONCLUSIONS

Blood‐based testing is expected to become the primary method for differential diagnosis of AD. In the long term, therapies for AD prevention may increase the need for blood‐based testing to screen for AD risk in the general population. Ocular testing offers a non‐invasive alternative to identify preclinical patients in different clinical settings (i.e., optometry and ophthalmology clinics), including patients with limited access to blood‐based testing. Given the frequency of routine eye exams in the aging population, the market for ocular diagnostics could be substantial.

Ocular technologies have yet to achieve the necessary standards for widespread adoption as an AD screening tool. While there are no obvious drivers behind the lack of definitive results for ocular technologies, we hypothesize that larger sample size studies that leverage tests and algorithms that are trained and validated with distinct datasets will elucidate whether the lack of definitive results is due to issues with analysis or source data. To play a role in the future AD diagnostic paradigm, two critical conditions must be met:

- Ocular technologies must meet the technical requirements (e.g., 90%–95% sensitivity, 75%–85% specificity) for preclinical AD screening and be comparable to blood‐based tests.
- Ocular technologies must demonstrate their clinical utility and actionability with available preclinical AD therapies.

Upon fulfillment of these criteria, additional investment in ocular screening may focus on:

- Evidence generation: studies demonstrating improved long‐term patient outcomes, reduced health‐care costs, and clinical utility of ocular screening.
- Automation support: development of applications to automate workflows and risk analyses.

It is important to note that some of the highlighted challenges that limit ocular adoption also slow the adoption of blood‐based biomarkers. Physicians have been hesitant to integrate AD screening tools into their clinical workups given the limited number of therapies available to act on any positive results. Blood tests for AD can also be expensive, ranging from $200 to > $1000, and are not currently reimbursed at rates to cover those costs.^54^, ^55^ Some patients may find blood draws invasive or they may be contraindicated based on other conditions. There are no point of care blood tests, as testing is typically done in a central or non‐local lab, which can take multiple days to return results. Additionally, while evidence supporting these new diagnostic modalities is growing, there remains limited information on the real‐world use of these tools. Continued investment and future scientific advances in this space will further erode the barriers to widespread adoption.

This work focused on the United States, as novel biotechnologies are often first available in the United States, and the region serves as the largest geographic market segment for most commercial players.^56^ In addition, constraining the scope of this work to one geographic market was necessary to ensure that the analysis was specific to a population, as market needs and dynamics vary significantly from country to country. Nonetheless, consideration of ocular biomarkers in a global context, particularly in countries with different regulatory infrastructures, is an important direction for future research and discussions. For example, some companies today are developing low‐cost ocular technologies that are meant to be applied in the developing world.^57^ Innovations like these will continue to emerge and will require further investigation on their potential global impact beyond the United States.

While there is still significant progress to be made, ocular technologies hold considerable promise as an alternative to blood‐based testing. With ongoing technological advancements, ocular biomarkers could become a scalable, accessible tool that can be integrated into routine eye exams, making early detection more feasible and improving outcomes for at‐risk individuals.

## CONFLICT OF INTEREST STATEMENT

All authors declare no conflicts of interest. Author disclosures are available in the supporting information.

## Supporting information



Supporting Information
